# Human SCARB2 Transgenic Mice as an Infectious Animal Model for Enterovirus 71

**DOI:** 10.1371/journal.pone.0057591

**Published:** 2013-02-25

**Authors:** Yi-Wen Lin, Shu-Ling Yu, Hsiao-Yun Shao, Hsiang-Yin Lin, Chia-Chyi Liu, Kuang-Nan Hsiao, Ebenezer Chitra, Yueh-Liang Tsou, Hsuen-Wen Chang, Charles Sia, Pele Chong, Yen-Hung Chow

**Affiliations:** 1 Institute of Infectious Disease and Vaccinology, National Health Research Institutes, Zhunan, Miaoli County, Taiwan; 2 Graduate Program of Biotechnology in Medicine, Institute of Molecular Medicine, National Tsing Hua University, Hsinchu, Taiwan; 3 Graduate Institute of Immunology, China Medical University, Taichung, Taiwan; Temple University School of Medicine, United States of America

## Abstract

Enterovirus 71 (EV71) and coxsackievirus (CVA) are the most common causative factors for hand, foot, and mouth disease (HFMD) and neurological disorders in children. Lack of a reliable animal model is an issue in investigating EV71-induced disease manifestation in humans, and the current clinical therapies are symptomatic. We generated a novel EV71-infectious model with hSCARB2-transgenic mice expressing the discovered receptor human SCARB2 (hSCARB2). The challenge of hSCARB2-transgenic mice with clinical isolates of EV71 and CVA16 resulted in HFMD-like and neurological syndromes caused by E59 (B4) and N2838 (B5) strains, and lethal paralysis caused by 5746 (C2), N3340 (C4), and CVA16. EV71 viral loads were evident in the tissues and CNS accompanied the upregulated pro-inflammatory mediators (CXCL10, CCL3, TNF-α, and IL-6), correlating to recruitment of the infiltrated T lymphocytes that result in severe diseases. Transgenic mice pre-immunized with live E59 or the FI-E59 vaccine was able to resist the subsequent lethal challenge with EV71. These results indicate that hSCARB2-transgenic mice are a useful model for assessing anti-EV71 medications and for studying the pathogenesis induced by EV71.

## Introduction

The epidemic of enterovirus 71 (EV71) infections occurring over the past 10 years in the Asia-Pacific region have caused serious public health concerns and highlight the necessity for the urgent development of the EV71 vaccine [1], [2], [3], [4], [5], [6], [7]. The clinical course has previously been to control EV71 infection and relies only on symptomatic treatment. EV71 is associated with HFMD and shows symptoms of persistent fever, herpangina, and lymphopenia [2], [8], [9]. The main severe symptom of EV71 is neural disorder, induced by inflammation in the central nervous system (CNS), leading to encephalitis and acute flaccid paralysis, pulmonary edema (PE), or hemorrhage, culminating in fatality, particularly in under 5 years old children [2], [8], [9], [10], [11].

Numerous animal models have been developed to study the pathogenesis of EV71 infection using the mouse-adapted strain of EV71 [12], [13], innate immunodeficient mice [14], or monkey models [15]. The intraperitoneal (i.p.) challenge of EV71 to adult mice caused no apparent clinical symptoms. Administration of mouse-adapted EV71 strain 4643 (Tainan/4643/98) to 1-d-old ICR mice caused hind limb paralysis (LP) and death within 2 wk of the challenge [12]. Following 1-d-old BALB/c and ICR mice infected with EV71 YN3 strain was also lethal [13]. A deficiency in type I and type II IFN receptors of the AG129 mouse caused neurological manifestations after infection with the non-mouse adapted EV71 strain (5865/SIN/00009; [14]. The EV71 BrCr strain, an original prototype of the genotype A strain from California [16], was demonstrated to induce neurological manifestations of tremor, ataxia, and brain edema, but no PE and cardiac failure in cynomolgus monkeys [15]. These models are not perfect for HFMD or for neuropathogenesis caused by EV71 in humans.

Activated lymphocytes may infiltrate into the infected CNS, attracted by secreted chemokines, and accumulate in the CNS, ultimately resulting in long-term neuropathology during viral infection [17]. In acute EV71 infection, massive IL-1β, IL-6, and TNF-α secretion was observed in the serum and cerebrospinal fluid of EV71-infected patients with PE and encephalitis, demonstrating a significant correlation between the pro-inflammatory cytokines and the severity of the disease [18], [19], [20], [21].

Human P-selectin glycoprotein ligand-1 (PSGL-1; [22] and human scavenger receptor class B, member 2 (hSCARB2; [23] have been identified as cellular receptors for EV71. PSGL-1, restrictively expressed in leukocytes, plays a role in binding leukocytes to endothelial cells and platelets and in the early stages of inflammation [24], [25], [26]. PSGL-1-Tg mice were generated but failed to enhance the diseases of clinical EV71 strains [27]. A type II glycoprotein of SCARB2 is expressed in many tissues, primarily in the limiting membranes of cell lysosomes and endosomes [28], [29]. Although mouse SCARB2 shares 85.8% homology to human SCARB2, it does not serve as a receptor for EV71 infection. Mapping study of the SCARB2 demonstrated that amino acids 142 to 204 from the human sequence is important for EV71 binding and infection [30]. We created a transgenic mouse expressing the hSCARB2 gene (hSCARB2-Tg) and studied the susceptibility of hSCARB2-Tg and the pathogenesis of EV71 infection. Infection of young (from 1-d-old up to 2-wk-old) hSCARB2-Tg mice with 4 clinical isolates of EV71 in which two B genotypes of EV71, E59 (B4) and N-2838 (B5) led to HFMD-like diseases, followed by neuropathogenesis induced by C genotypes of EV71 such as 5746 (C2) and N-3340 (C4) and even CVA16 leading to lethal LP. Replication of EV71 in the transgenic mice, coupled with induced pro-inflammatory cytokines, resulted in T lymphocyte infiltration in the tissues and showed a correlation to the severity of EV71-mediated pathogenicity. Finally, pre-immunization of the EV71 vaccine in hSCARB2-Tg mice demonstrated the cross-protective immunities preventing lethal EV71 infection.

## Results

### Generation and Screening of hSCARB2-transgenic Mice

**Figure 1:**
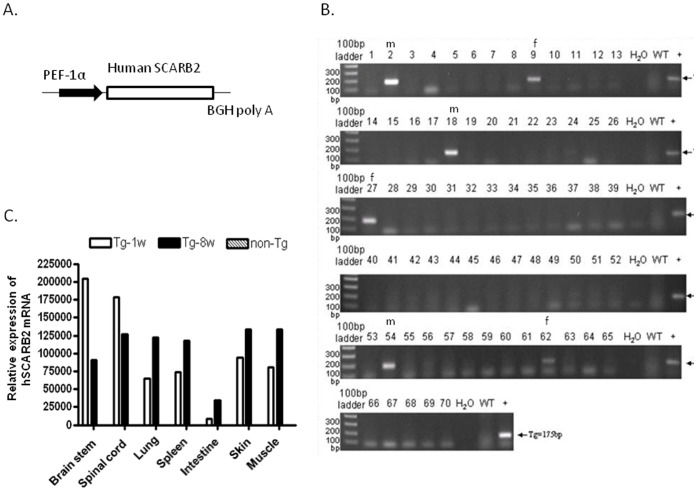
Creation and screening of hSCARB2-Tg mice. (**A**) The human SCARB2 gene construct was used to create transgenic mice. The human SCARB2 was cloned under the hEF1α promoter and BGH poly A tail to obtain a pEF-1α-hSCARB2 expression vector, which was used for embryo microinjection. (**B**) Genomic PCR of the tail DNA of hSCARB2-Tg mice using primer set 1 (Table S1) was set to screen for the presence of the hSCARB2 transgene. The size of PCR and RT-PCR products is 175 bp. (**C**) Quantitative RT-PCR analysis of RNA using primer set 2 (Table S1) was extracted from different tissues of 8-wk-old and 1-wk-old transgenic mice and 8-wk-old non-transgenic mice as control to quantify hSCARB2 expression was performed. β-Actin gene expression in each tissue was used as the internal control. Each normalized 2^Ct^ value was ratio to the value from the mean of 2^Ct^ obtained from the muscle tissues of non-transgenic mice. A schematic representation of the hSCARB2 gene expression and the statistical average from 7 mice per group was shown.

To create an hSCARB2 transgenic mouse, full-length codon-optimized hSCARB2 cDNA was cloned into a pEF1α vector (Fig. 1A) and the resulting construct was microinjected into C57BL/6 embryos to generate founder hSCARB2-Tg mice. Screening of the founder was conducted by genomic PCR using primers specific to the hSCARB2 gene to amplify a 175 bp fragment. Six founder mice–No. 2 (male, m), No. 9 (female, f), No. 18 (m), No. 27 (f), No. 54 (m), and No. 62 (f)–were identified to carry the hSCARB2 transgene (Fig. 1B). The founders were then cross-mated with wild-type C57BL/6 mice to produce F1 progenies. The hSCARB2-positive F1 mice were inbred and screened by genomic PCR using the primer set 1 (Table S1) until the homozygous hSCARB2 transgenic line was obtained. Since the available antibodies could cross-react human and mouse SCARB2 (data not shown). The expression of hSCARB2 in the tissues of transgenic mice, compared to the tissues of non-Tg, was detected by real-time RT-PCR using the primer set 2 (Table S1) which specifically targeted at the 126 nucleotide (forward) and the 389 nucleotide (reverse) of the human SCARB2 sequence to distinguish the human and mouse SCARB2 cDNA (Fig. S1). The level of hSCARB2 expressed in the tissues of young (1-wk-old, 1 w) and adult (8 w) hSCARB2-Tg mice was comparably detected but was lower in the intestine. No signal of hSCARB2 expression was found in the tissues of adult non-Tg C57BL/6 mice (Fig. 1C).

### B genotype of EV71 Induces HFMD-like and Neurological Diseases in hSCARB2-transgenic Mice

**Figure 2:**
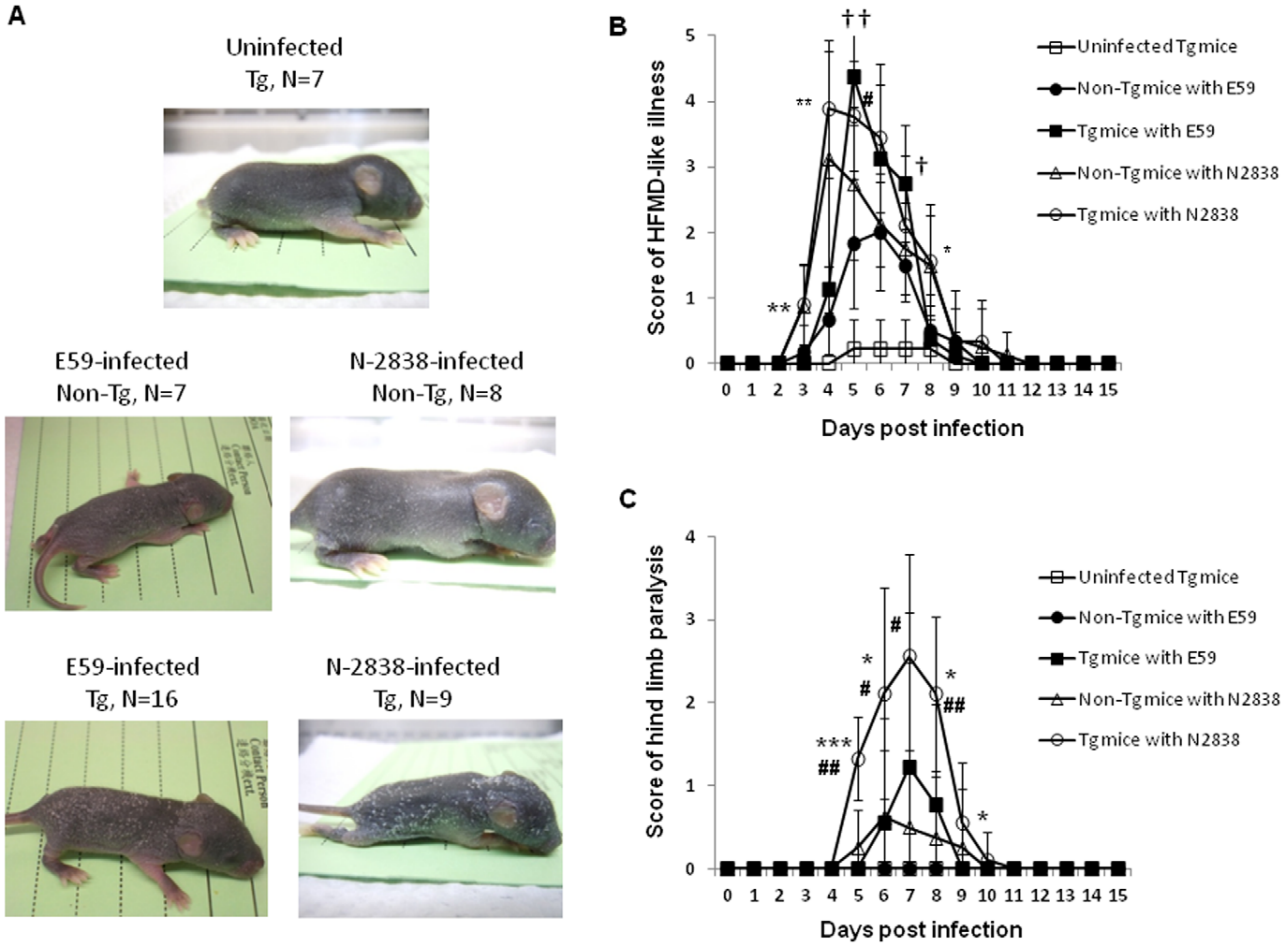
Disease symptoms in hSCARB2-Tg mice infected with the B genotype of EV71. (**A**) 1-d-old hSCARB2-Tg mice were injected s.c. with 1×10^7^ pfu of E59 (B4) or the N-2838 (B5) strain. As a negative control, 1-d-old hSCARB2-Tg mice were injected with s.c. with a VP-SFM medium, including non-Tg mice infected with 1×10^7^ pfu of E59 or N-2838. The mice were monitored daily to (**B**) score the HFMD-like syndrome and (**C**) the CNS-like hind limb paralysis, followed the criteria described in the Materials and Methods section. Data are representative of the mean scores obtained from the individual number of (N) mice per group. One-way anova with Kruskal-Wallis test was used for statistical analysis and the error bar of each group was included. Significant difference of Tg mice infected with N-2838 vs. E59 was shown as *: p<0.05, **: p<0.01, and ***: p<0.001, the Tg mice vs. non-Tg infected with E59 was shown as †: p<0.05 and ††: p<0.01, and the Tg mice vs. non-Tg infected with N-2838 was shown as #: p<0.05 and ##: p<0.01.

Phylogenetic analysis of the VP1 sequence showed that 3 distinct EV71 genotypes (A, B, and C) were identified [16]. One-day-old Tg and non-Tg mice were challenged with E59 (B4) and N2838 (B5) strains subcutaneously (s.c.) and their behavior was monitored regularly. Visible hair loss associated with scurf (HLS) was observed in all Tg mice infected with E59 and N2838 on Day 6 of post-infection, whereas mild HLS was observed in non-Tg mice infected with E59 or N-2838, compared to culture medium-administrated Tg mice (Fig. 2A and Table 1). HLS observed in hSCARB2-Tg mice corresponds to the rash (lesions) that appears on the palms and soles, and with vesicles in the mouth of EV71-infected children [31]. Scoring of the HFMD-like syndrome showed HLS severity to be associated with hSCARB2 expression in mice, in which E59 and N-2838 induced higher HLS symptom in Tg mice compared to the non-Tg mice infected with the respective pathogens. N-2838 seems eliciting more virulence than E59 in the induction of HLS in both Tg and non-Tg mice (Fig. 2B). A CNS-like illness involving LP was also found in Tg mice administrated with both B genotypes of EV71 after 6 d of challenge; Tg, not non-Tg mice, exhibited severe LP (Fig. 2C). However, the older (2-wk-old and up) Tg mice infected with EV71 E59 or N-2838 showed mild HFMD-like and neurological symptoms and finally recovery (data not shown).

**Table 1:** HFMD and CNS diseases induced by different clinical isolates of EV71 and CVA16 in hSCARB2-Tg mice.

Virus strains (genotype)	HFMD		CNS			
			Limb paralysis (%)		Death (%)	
	Tg	Non-Tg	Tg	Non-Tg	Tg	Non-Tg
**E59(B4)**	100(16/16)	57.1(4/7)	18.8(3/16)	14.2(1/7)	0(0/16)	0(0/7)
**N-2838(B5)**	100(9/9)	75(6/8)	44.4(4/9)	12.5(1/8)	0(0/9)	0(0/8)
**5746(C2)**	#Nd	#Nd	100(10/10)	57.1(4/7)	100(10/10)	0(0/7)
**N-3340(C4)**	Nd	Nd	100(7/7)	100(7/7)	100(7/7)	0(0/7)
**CVA16**	Nd	Nd	100(8/8)	100(6/6)	100(8/8)	100(6/6)

1-d-old Tg and non-Tg mice were injected s.c. with 1×10^7^ pfu of E59 or N-2838, and 7-d-old Tg and non-Tg mice were injected with 3×10^4^ pfu of 5746 or N-3340 EV71 strains or 3×10^5^ pfu of CVA16. The signs of HFMD and CNS diseases were monitored. The incidence of severe disease (the number of animals scored >3) on day 10 post challenge was shown as the percentage rate (number of mice with disease per total number of tested mice).

# disease was not detected.

### C genotype of EV71 Induces Pathology in hSCARB2-trangenic Mice

**Figure 3:**
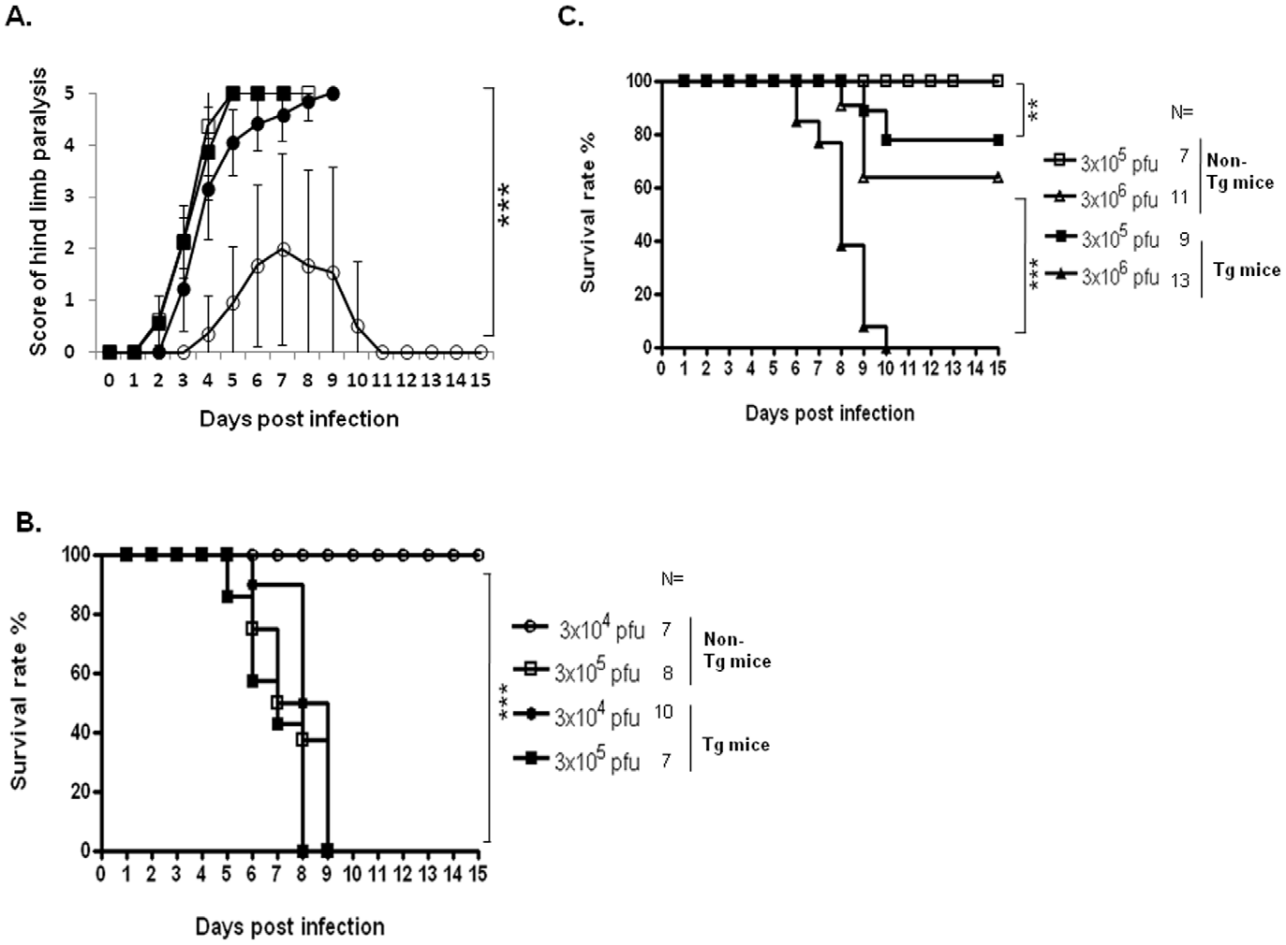
Lethality and neurological symptoms in mice infected with the C genotype of EV71. Scoring of (**A**) CNS-like hind limb paralysis and (**B**) survival rate in 7-d-old hSCARB2-Tg and non-Tg mice injected with various dose of 5746 were assessed following the criteria described in the Materials and Methods section. One-way anova with Kruskal-Wallis test was used to analyze the statistic difference of hind limb paralysis observed in Tg mice s.c. infected with 3×10^4^ (•) or 3×10^5^ (▪) pfu of 5746 vs. non-Tg mice infected with 3×10^4^ (○) or 3×10^5^ (□) pfu of 5746. (**C**) Daily survival rate of 14-d-old hSCARB2-Tg mice s.c. infected with 3×10^5^ (▪) or 3×10^6^ (▴) pfu of 5746 or non-Tg mice infected with 3×10^5^ (□) or 3×10^6^ (Δ) pfu of 5746 were monitored. Logrank test used to analyze the statistic difference of survival rate of 5746-infected Tg vs. non-Tg mice and the error bar of each group was included. The number (N) of mice per group was shown.

Because the epidemic in Taiwan has shown that most strains belong to the C2 genotype and a minority to the B genotype [32], [33], it prompted us to investigate the virulence of the C genotype of EV71 in hSCARB2-Tg mice. In 7-d-old Tg mice, Tg mice challenged with a low dose (3×10^4^ pfu) of 5746 (C2) showed severe LP resulting in all death on Day 9, compared to non-Tg mice elicited by only mild LP, from which they recovered by Day 11 (Fig. 3A). The challenge with a low doses of 5746 resulted in 100% death in Tg mice (10/10 died) by Day 9 of post-infection, but all non-Tg mice survived (0/7 died; Fig. 3B). Tg mice challenged with a moderate dose (3×10^5^ pfu) of 5746 began to die on Day5 and then completely died (7/7) by Day 8 of the post-viral challenge. Non-Tg mice with the same dose began to die on day 6 and were delayed to completely die by Day 9 (8/8; Fig. 3B).

When 14-d-old hSCARB2-Tg mice were challenged with a high dose (3×10^6^ pfu); they all died (13/13) by Day 10, whereas approximately 22% (2/9) died with a moderate dose (3×10^5^ pfu). In the comparison, only 36% of non-Tg mice (4/11) died with a high dose and all non-Tg mice survived (0/7) with a moderate dose (Fig. 3C). In 21-d-old mice receiving s.c. 3×10^6^ pfu of EV71, we observed that 0% died (0/7) by Day 10 post-inoculation, and all the non-Tg mice survived (0/7) (Fig. S2).

We examined other C genotypes of EV71 and CVA16 in Tg mice. Using hSCARB2 as a receptor for CVA16 infection has been reported [23]. The result showed that 5746 (C2) and N3340 (C4) strains of EV71 and coxsackievirus A16 (CVA16) induced severe CNS diseases, resulting in death in newborn Tg mice, parallel to the major HFMD diseases induced by E59 (B4) and N2838 (B5; Table 1). Newborn non-Tg mice were less sensitive to B genotypes of EV71 (E59 and N-2838) than Tg mice that induced HFMD and non-lethal LP diseases. Additionally, newborn non-Tg mice also elicited less susceptibility to two strains of C genotypes of EV71, 5746 and N-3340, that induced severe lethal LP compared to newborn Tg mice. CVA16 is closely related to EV71, both in genetic and amino acid sequence. In contrast to EV71-related HFMD complicated with neurological diseases, CVA16 responds HFMD disease but rarely associates with neurological complications in children. Interestingly, CVA16 also induced a severe LP disease causing complete death (100%) in our newborn Tg mice but partial death (83.3%) in non-Tg mice (Table 1). We conclude that 2 distinct pathology tropisms of EV71 exist in hSCARB2-Tg mice; a group induces lethal CNS diseases such as C2 and C4 genotypes of EV71 and CVA16, and a group induces HFMD diseases, including B4 and B5 genotypes of EV71.

### Pathology of EV71-induced Complications in hSCARB2-transgenic Mice

**Figure 4:**
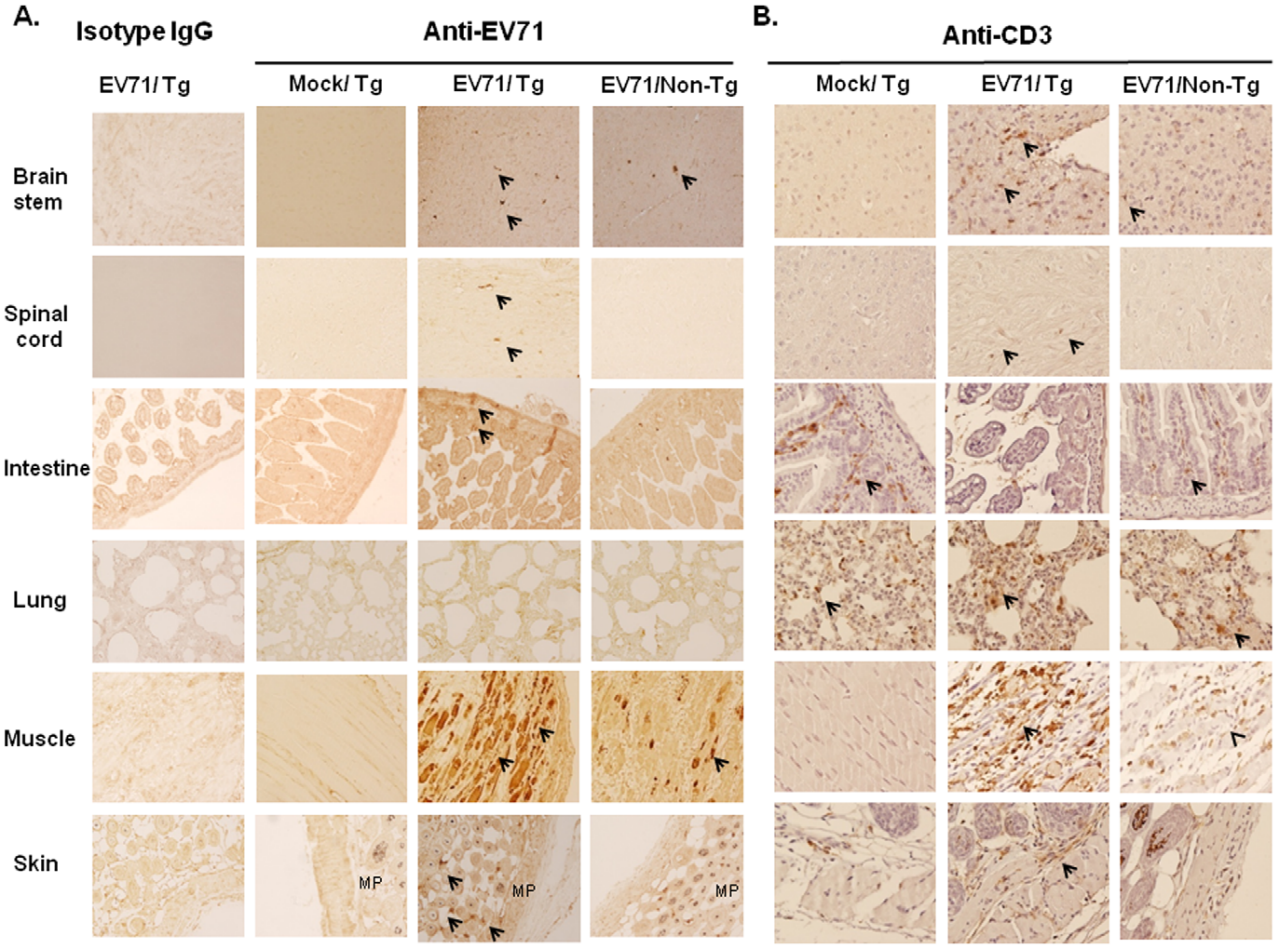
In situ EV71 distribution in hSCARB2-Tg mice. Seven-day-old hSACRB2-Tg and non-Tg mice infected with 3×10^4^ pfu of EV71 5746 s.c. were sacrificed on Day 7 post-infection. Uninfected hSCARB2-Tg mice were used as the negative control. Waxed sections of the brainstem, spinal cord, intestine, lung, biceps femoris muscle, and lower back skin were prepared and IHC stained with (**A**) Mab979 antibody or isotype mouse IgG and (**B**) the anti-CD3 antibody. All pictures were taken at 200X magnification. Viral particles or T lymphocytes in the sections are indicated with arrows. The melanin pigments (MP) pale-stained by Mab979 antibody in the section of skin tissue was observed.

Immunohistochemical (IHC) and real-time RT-PCR analysis were performed to detect EV71 in the tissues of hSCARB2-Tg and non-Tg mice on Day 7 post-infection, while peaked the HFMD (Fig. 2) and CNS (Fig. 3) symptoms. Severe decomposition of muscular fiber was observed in EV71 5746-infected Tg mice, but mild destruction in non-Tg mice and microvilli in the inner layer of the small intestine of Tg mice was completely destroyed by EV71 infection (Fig. 4). Signals for VP1 were detected by IHC in the brainstem, spinal cord, intestine, biceps femoris muscle, and the dermis of the lower back skin of infected Tg mice, whereas only a pale signal was detected in these tissues except the muscle of non-Tg mice and only background staining was observed in uninfected Tg mice. However, signals in the lung of infected Tg or non-Tg were not detectable (Fig. 4A). Because of lower sensitivity and no quantification of IHC analysis, we further detected EV71 in the infected mice using RT-PCR.

**Figure 5:**
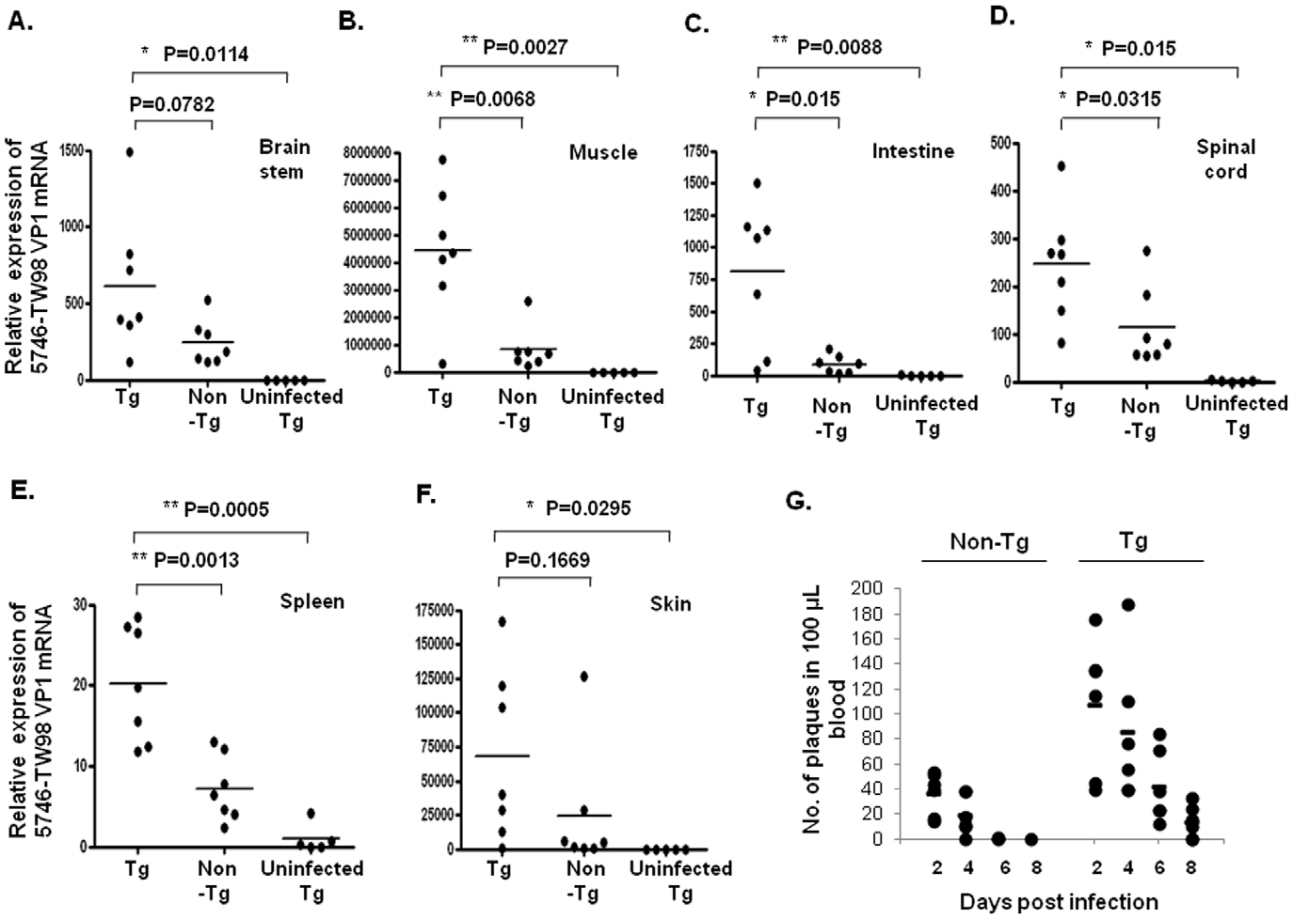
Viral distribution in the tissues and organs of EV71-infected mice. Seven-day-old hSCARB2-Tg or non-Tg mice were injected with 3×10^4^ pfu of 5746 s.c. and on Day 7 post-viral infection, RNA was extracted from the brainstem (**A**), muscle (**B**), intestine (**C**), spinal cord (**D**), spleen (**E**), and skin (**F**) of the mice and then subjected to quantitative RT-PCR analysis using primers specific to the VP1 region. β-Actin gene expression in each tissue was used as the internal control. A schematic representation of the VP1 gene expression and the statistical average from 7 mice per group is shown. (**G**) Peripheral blood was collected from the individual mice on Day 2, Day 4, Day 6, and Day 8 post-infection and then subjected to an immune plaque assay described in the Materials and Methods section. The results are presented as the individual number of plaques, and the calculated mean in 100 µl of blood was obtained from 5 mice per group. Unpaired student *t* test with Welch’s correction was used for statistical analysis.

To quantify the viral load of EV71 5746 strain in the infected hSCARB2-Tg mice, real-time RT-PCR of EV71 VP1 mRNA region in the different tissues of Tg and non-Tg mice on Day 7 post-EV71 5746 infection (while peaked the LP disease) was performed. EV71 was detected in the brainstem (Fig. 5A) and spinal cord (Fig. 5D), the viral load in the spinal cord of Tg mice being significantly higher than in non-Tg mice. Massive viral load in the muscle was observed in Tg mice, but not in non-Tg mice (Fig. 5B). EV71 in the intestines increased in Tg mice, but was basal in non-Tg mice (Fig. 5C). The presence of EV71 was also observed in the spleen (Fig. 5E), the site of immune response, and in the circulating blood (Fig. 5G), where more viruses were detected in Tg mice than in non-Tg mice. However, the viral load in the skin of Tg mice was not significantly higher than in the skin of non-Tg mice due to individual sample error (Fig. 5F). These results support severe neurological disease (Fig. 3) observed in EV71-infected Tg mice caused by the accumulation of EV71 in the CNS and the muscle tissues.

### Induction of Pro-inflammatory Mediators Linking Infiltrated Lymphocytes in EV71-infected hSCARB2-transgenic Mice

**Figure 6:**
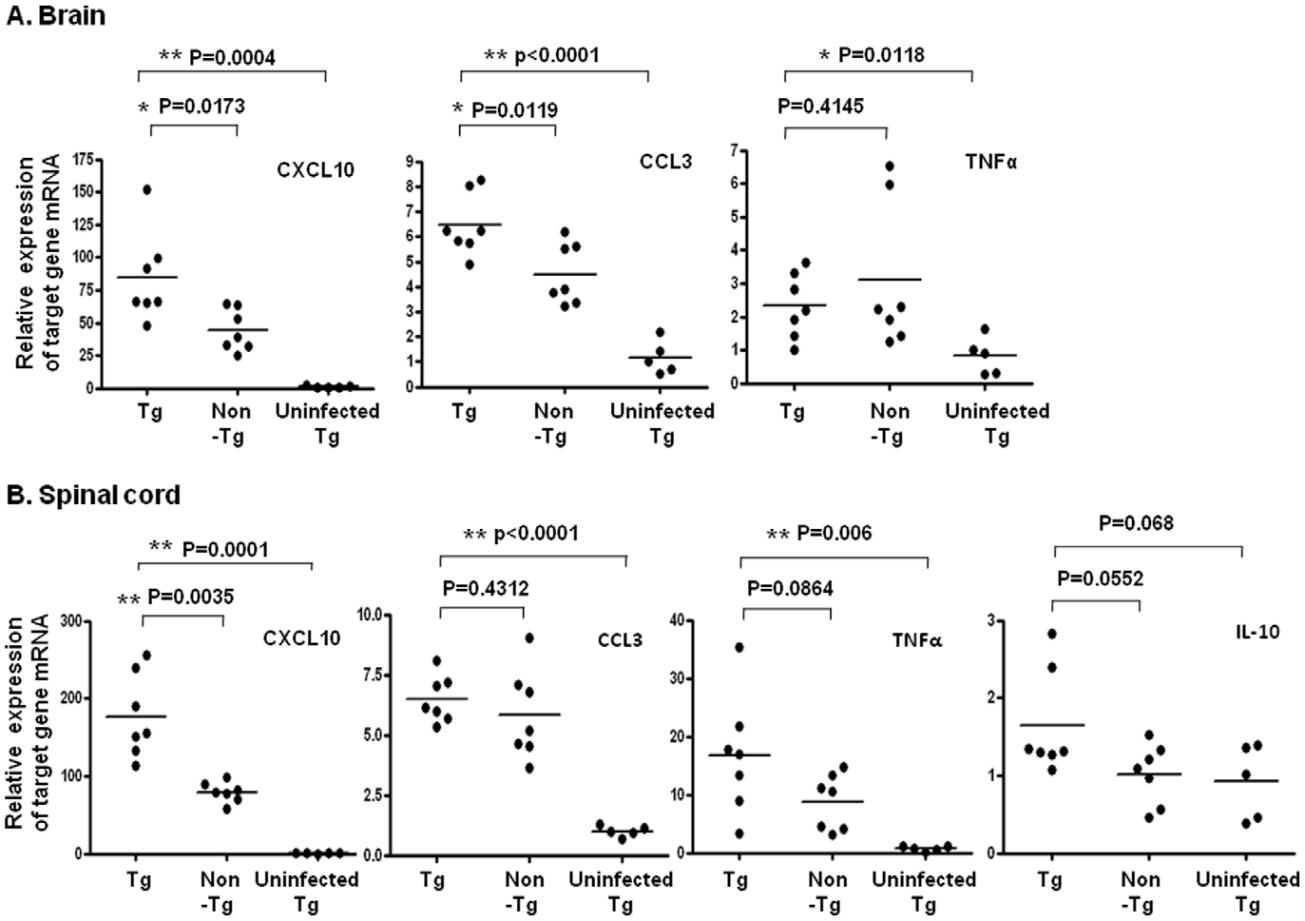
Expression of pro-inflammatory cytokines in the CNS compartments of EV71-infected mice. After a 7 d challenge of hSCARB2-Tg and non-Tg mice with 3×10^4^ pfu of 5746 s.c., RNA was extracted from the brainstem (**A**) and spinal cord (**B**) and quantitative RT-PCR analysis was conducted to quantify CXCL10, CCL3, TNF-α, and IL-10 genes. hSCARB2-Tg mice that received no EV71 were set as the negative control. The number of PCR cycles required for fluorescent detection of target genes was calculated and presented as the relative expression after normalization with the internal control of β-actin expression from the same tissue. A schematic representation of the target gene expression and the statistical average from 7 mice per group is shown. Unpaired student *t* test with Welch’s correction was used for statistical analysis.

EV71 patients with encephalitis associated with PE have a higher mortality rate (64.3%) than patients only generated encephalitis (26.3%; [8], [34]). PE might be caused by increased pulmonary vascular permeability resulting from brainstem lesions caused by the excessive release of IL-6, TNF-α, and IL-1β [18], [19]. PE was not observed in EV71-infected hSCARB2-Tg mice. Therefore, we measured the expression of pro-inflammatory mediators in EV71-infection hSCARB2-Tg mice using real-time RT-PCR. We found enhanced expression of CXCL10 and CCL3 in the brainstem of Tg mice, compared to infected non-Tg mice (Fig. 6A). However, TNF-α expression was elevated in infected Tg and in non-Tg mice (Fig. 6A). The expression of CXCL10 was higher in Tg mice than in non-Tg mice, whereas CCL3 and TNF-α increased in both Tg and non-Tg mice in the spinal cord (Fig. 6B). Although IL-10 slightly increased in Tg mice, no statistical difference existed (Fig. 6B). We also measured these mediators in the peripheral tissues. CXCL10, TNF-α, and IL-6 expression were upregulated in the muscles of both Tg and non-Tg mice, compared to uninfected mice (Fig. S3A). Increased CXCL10 and CCL3 were observed in the spleen of infected Tg and non-Tg mice (Fig. S3B). However, we did not detect these mediators in the skin and lung. These results demonstrated the upregulation of chemokines in the CNS and peripheral tissues of Tg mice corresponding to EV71 infection was significant (Infection vs. uninfection), but they could not completely interpret the detail mechanism of inflammatory chemokines-mediated severe EV71 pathogenesis in Tg but mild in non-Tg mice. Because inflammatory lymphocytes can be recruited into the CNS by secreted chemokines and cause neuropathology during viral infection [17], we further examined the infiltrated T lymphocytes in the infected tissues using IHC staining with the anti-CD3 antibody. We found T lymphocytes to be accumulated in the brainstem, spinal cord, lung, skin, and major muscles of infected Tg mice, but few were found in infected non-Tg mice, compared to uninfected mice (Fig. 4B). Because of microvilli destruction, we did not find the T lymphocytes in the intestine of Tg mice, but found some in the normal intestine of uninfected Tg and few in the mild impaired intestine of non-Tg mice (Fig. 4B). We concluded that CXCL10 (brain and spinal cord tissues) and CCL3 (brain tissue) were upregulated in response to EV71 infection in hSCARB2-Tg, but less in non-Tg mice, accompanied with chemo-attraction of T lymphocyte infiltration.

### B4 Strain of EV71 Immunization Cross-protects Mice from C2 Strain of the EV71 Challenge

**Figure 7:**
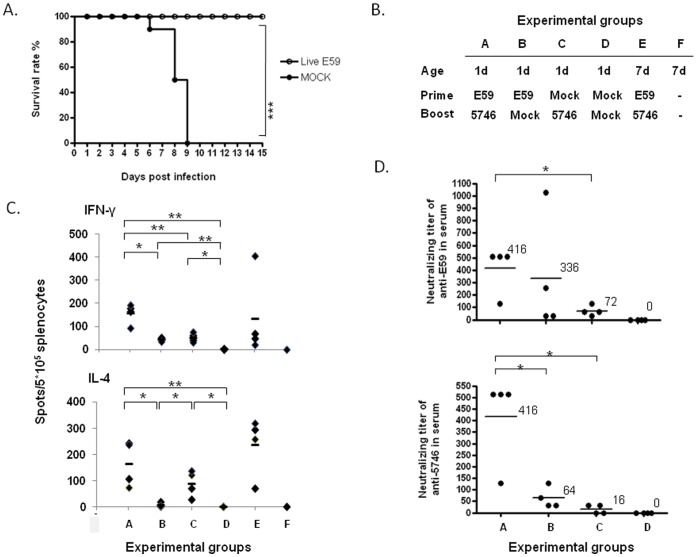
Cross-protection of hSCARB2-Tg mice against the 5746 challenge by pre-immunization with live E59. (**A**) Surviving 1-d-old hSCARB2-Tg mice were primed with 1×10^7^ pfu of live E59 s.c. (○) or medium (mock) (•) and then challenged with 3×10^4^ pfu of 5746 s.c. after 7 d. Twelve mice in the live E59 pre-immunized group and 10 mice in the mock-treated group were examined. Logrank test was used for statistical analysis. (**B**) Group of transgenic animals received immunization and/or challenge of EV71 was illustrated. One-day-old hSCARB2-Tg mice were immunized with 10^7^ pfu of live E59 or mock and then challenged on Day 21 of birth with 3×10^4^ pfu 5746 or mock. In another group, 7-d-old hSCARB2-Tg mice immunized with E59 and challenged on Day 21 of birth with 5746 was included. (**C**) We harvested 5×10^5^ splenocytes from individual mice on Day 28 of birth and subjected them to the IFN-γ and IL-4 ELISPOT assays that were described in the Materials and Methods. Data are representative of the results derived from 2 independent experiments, each with 4 mice per group. (**D**) Serum samples were also collected from individual mice while sacrificed as following the schedules described in (**B**) and were assayed for the titer of anti-E59 and anti-5746 neutralizing antibodies described in the Materials and Methods section. The results were expressed as titers for each test sample. Bars correspond to the mean titers for each experimental group. Unpaired student *t* test with Welch’s correction was used for statistical analysis of (**C**) **and (D**).

Finally, we evaluated whether the Tg mice could serve as a model for evaluating the effectiveness of anti-EV71 medications. Pre-immunization of 1-d-old hSCARB2-Tg mice with 1×10^7^ pfu of live E59, followed by the challenge with 10 times the lethal dose (3×10^5^ pfu) of 5746 on Day 7, resulted in 100% survival in pre-immunized Tg mice, compared to 100% mortality in unimmunized Tg mice (Fig. 7A). Pre-immunization of live E59 induced HLS syndrome as early as on day 4 post infection (p.i.) and then peaked on day 6 p.i. in Tg mice was observed (data not shown) as usual as shown in Fig. 2. Adaptive immunity specific to EV71 was induced in the pre-immunized mice, as evidenced by the higher expression of splenocytic IFN-γ and IL-4 observed in 1-d- and 7-d-old hSCARB2-Tg mice immunized and challenged with E59 and 5746, respectively, compared to mice only immunized or only challenged with the virus (Fig. 7B). Neutralizing antibodies against E59 and 5746 were significantly raised in the serum of mice received both prime and challenge, compared to only prime or challenge of EV71 (Fig. 7C), supporting the protective immunities generated in the immunized newborn Tg mice.

**Figure 8:**
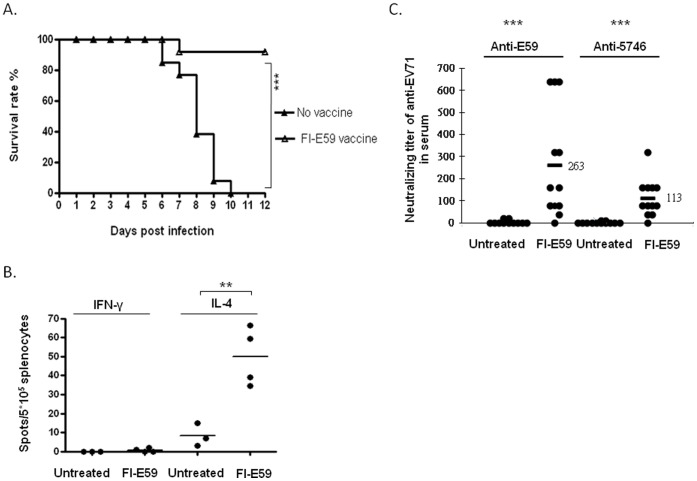
Protection of hSCARB2-Tg mice from 5746 challenge by formalin-inactivated E59 vaccine. Survival of hSCARB2-Tg mice intramuscularly pre-immunized twice with (Δ) or without (▴) 1 µg of FI-E59 on Day 1 and Day 8 of birth prior to being challenged with 3×10^6^ pfu of 5746 s.c. Twelve mice in the FI-E59 vaccine-treated group and 13 mice in the untreated group were examined. Logrank test was used for statistical analysis. (**B**) After the immunization of 1 µg of FI-E59 on Day 1 and Day 8 of birth, mice were sacrificed on Day 14 of birth and the spleens were pooled from 3 individual mice and the isolated 5×10^5^ splenocytes per well were subjected to the IFN-γ and IL-4 ELISPOT assays. Data are representative of the results derived from 2 independent experiments, each with 4 mice per group. (**C**) Serum samples collected from individual mice on Day 14 of birth prior to being challenged with 3×10^5^ pfu of 5746 s.c. were assayed for the titer of anti-E59 and anti-5746 neutralizing antibodies described in the Materials and Methods section. The results were expressed as titers for each test sample. Bars correspond to the mean titers for each experimental group. Unpaired student *t* test with Welch’s correction was used for statistical analysis of (**B**) and (**C**).

Since B4 genotype of clinical isolate EV71 E59 strain was outbreak in 1998 in Taiwan [3], [8] and there is no certified EV71 vaccine available in the world, every party working on EV71 vaccine development collaborates with the regulatory authority in their own country. We have been approved by Taiwan Food and Drug administration to produce formalin-inactivated EV71 E59 (FI-E59) vaccine candidate containing sub-microgram of viral proteins formulated with alum adjuvant and use in phase I clinical trial [35]. Mice primed and boosted with 1 µg of the formalin-inactivated EV71 (FI-E59) vaccine on aluminum phosphate on Day 1 and Day 8 after birth followed by the challenge with 3×10^6^ pfu of 5746 on Day 14 showed 90% protection compared to Tg mice without vaccine pre-immunization (Fig. 8A). Secretion of splenocytic IL-4 was observed on Day 14 of birth of mice pre-immunized with FI-E59 vaccine while significant IFN-γ spots were not recorded (Fig. 8B), indicating the induction of Th2-mediated humoral immunity by formalin-inactivated vaccine. Supporting result of neutralizing antibodies against E59 and 5746 were detected in the serum of mice pre-immunized with FI-E59 vaccine on Day 14 of birth before being challenged with 5746 (Fig. 8C).

**Figure 9:**
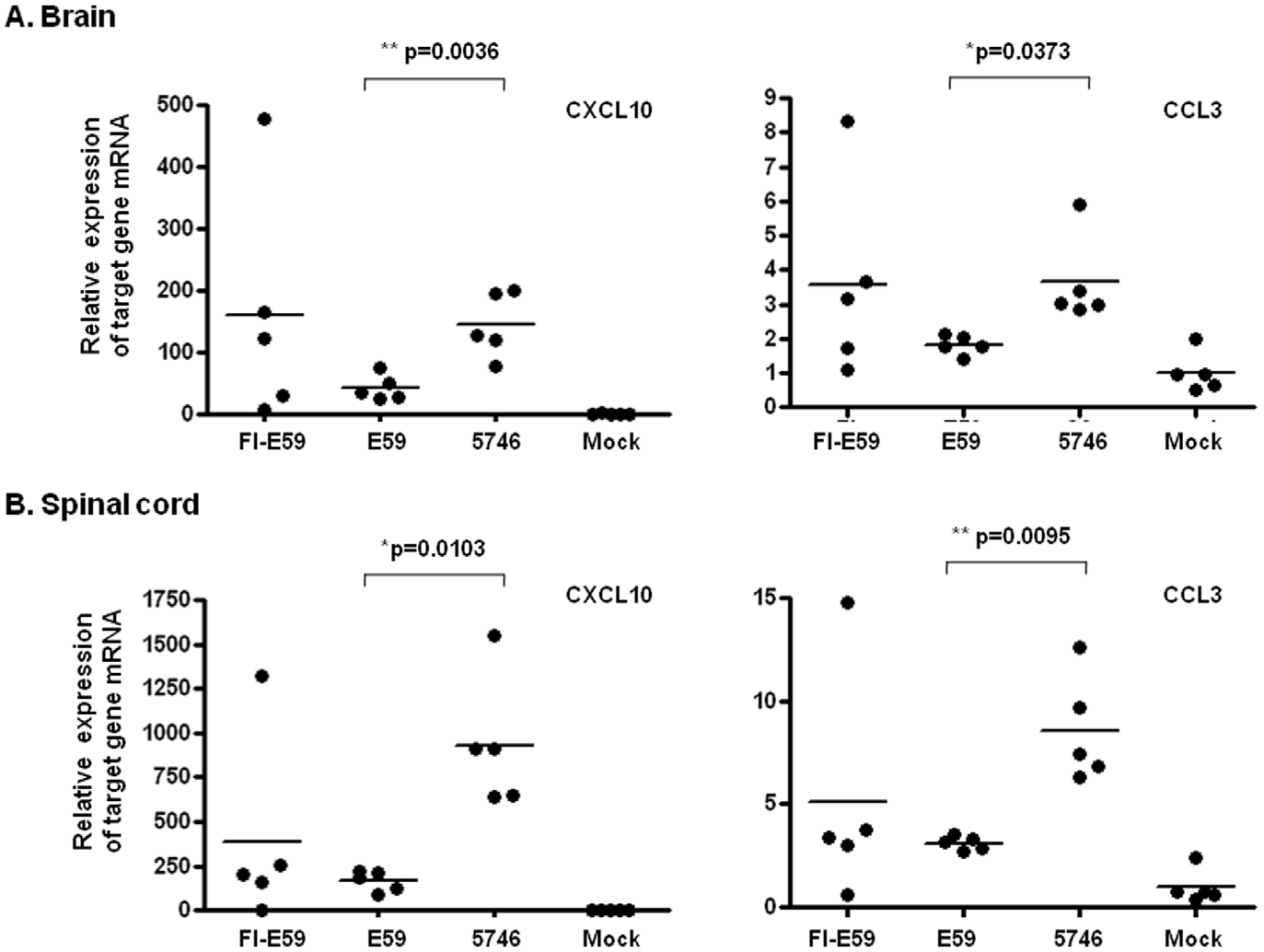
Expression of CXCL10 and CCL3 in the CNS of different genotype of EV71- or FI-E59 vaccine-inoculated mice. After a 7 d injection s.c. of 1-day-old hSCARB2-Tg with 1×10^7^ pfu of E59, 3×10^4^ pfu of 5746, or 1 µg of FI-E59, RNA was extracted from the brainstem (**A**) and spinal cord (**B**) and quantitative RT-PCR analysis was conducted to quantify CXCL10 and CCL3 genes. hSCARB2-Tg mice that received no EV71 (mock) were set as the negative control. The relative expression of chemokines was calculated as described in the legend of Fig. 6. The statistical average from 5 mice per group is shown. Unpaired student *t* test with Welch’s correction was used for statistical analysis.

Since CXCL10 and CCL3 were significantly elevated in the CNS compartments of the hSCARB2-Tg mice infected with 5746 (C2) and were concluded to be involved in EV71-mediated neurologic pathogenesis (Fig. 6). We further investigated whether this upregulation was observed in the inoculation of live E59 (B4) which elicited mild to moderate CNS diseases in transgenic mice or FI-E59 vaccine. After a 7 d challenge of hSCARB2-Tg mice with E59 vs. 5746, both CXCL10 and CCL3 were not significantly increased in E59-inoculated brainstem and spinal cord tissues, compared to 5746-treated tissues (Fig. 9). Interestingly, the level of CXCL10 and CCL3 in FI-E59 vaccine-inoculated CNS tissues was also upregulated (Fig. 9). These results indicate that the secretion of CXCL10 and CCL3 correlated to the severity of neurologic pathogenesis induced by EV71 is confirmed. The immunity induced by the live B4 genotype of EV71 or the formalin-inactivated vaccine is able to cross-protect against the challenge by the C2 genotype of EV71 in young hSCARB2-Tg mice. However, the potential neurological side effect induced by formalin-inactivated vaccine need to be accounted. Our hSCARB2-Tg mice not only serve as an animal model for studying EV71-mediated pathology but also are also useful for evaluating anti-EV71 vaccines.

## Discussion

We successfully generated an hSCARB2-expressing transgenic animal. Under the challenge of newborn hSCARB2-Tg mice with the E59 strain, we observed the early syndrome of HFMD, followed by progressive CNS-mediated LP (Fig. 2). This is the first report of inducing the natural strain of EV71 (B4 and B5)-mediated HFMD-like syndrome in transgenic mice. Other newborn (1 d- to 1-wk-old) mice only showed neurological pathology when infected with the natural non-existing mouse-adapted EV71 [12], [20], [36], [37], [38], in the natural strain of EV71 in type I/II interferon-deficient newborn mice [14], or in cynomolgus monkeys [15]. We demonstrated that hSCARB2-Tg mice elicit more susceptibility than the reported newborn mouse models for natural EV71 infection, resulting in severe HFMD (Fig. 2) and neurological diseases, even occurring in 3-wk-old Tg mice (Fig. 3). Despite the muscle-tropic EV71 viremia in the reported model that differ from the CNS-tropism of EV71 infection in humans, hSCARB2-Tg mice were characterized as bi-pathological tropism in the CNS and peripheral sites, including muscle, intestine, and skin (Fig. 5).

CXCL10 is expressed primarily by ependymal cells, astrocytes, and microglia [39], to aid in defense during acute diseases by attracting activated T and NK cells into the CNS [40], [41], [42]. We found that higher CXCL10 observed in the CNS, muscle, and spleen of EV71-infected Tg mice (Fig. 6) that might correlate the T cell infiltration (Fig. 4B) which also corresponds to the clinical findings where CXCL10 increased in patients with EV71 [43]. Higher expression of CCL3 observed in the CNS of Tg mice infected with EV71 (Fig. 6) may also contribute to the recruitment of granulocytes (neutrophils) during inflammation in the brain [44], [45], [46]. In comparison of 5746-infected Tg vs. non-Tg mice, Tg mice showed severe LP and more virus loads resulted in death, but there was no significant difference in IL-6 and TNF-α expression (Fig. 6 and Fig. S3). The induction of IL-6 in cerebrospinal fluid (CSF) contributed to the severe pathogenesis in EV71 PE patients was reported [19]. However, the contradictory results had been shown that EV71 cases involving brainstem encephalitis plus PE had a significantly lower cellular IL-6 and TNF-α expression, compared to EV71 patients with CNS involvement and uncomplicated EV71 cases [47]. Thus, the role of IL-6 and TNF-α in EV71-induced pathogenesis in this model should be further investigated. Increased IL-10 in the plasma has been recorded in children with EV71 PE [34], but was marginally increased only in the spinal cord of Tg mice, compared to non-Tg and uninfected Tg mice (Fig. 6B), probably because of the degradation in mouse serum. We conclude that release of CXCL10 and CCL3 in response to EV71 infection in hSCARB2-Tg mice strongly correlates with pathological responses that are similar in EV71-infected patients.

The differential severity of HFMD disease induced by genotypes of B and C of EV71 infection in children was not reported [2], [8], [9]. However, the genotypes of B and C of EV71 contributing to differential virulence judged by CNS diseases in hSCARB2-Tg mice and humans [2], [8], [9], [10], [11] were observed. A high dose (1×10^7^ pfu) of E59 (B4) and N2838 (B5) induces HFMD-like but mild neurological diseases, whereas receiving a lower dose (3×10^5^ pfu) of 5746 (C2) and N3340 (C4) results in severe neurological paralysis accompanied by lethality (Fig. 2, Fig. 3, and Table I). The amount of viral loads distributed in the tissues might correspond to the virulence induced by the different genotypes of EV71. Massive viremia was detected in the CNS and peripheral tissues of 5746-challenged Tg mice and led to lethal neurological diseases, but less viral loads were observed in the tissues of non-Tg mice (Fig. 5) which correlated to the induction of mild symptoms (Fig. 3). B genotype of EV71 (E59 or N2838) induced severer HFMD and LP diseases in Tg mice than in non-Tg mice, and CVA16 induced severe CNS disease in Tg compared to non-Tg mice (Table 1), indicating the increase of susceptibility of mice infected with EV71 and CVA by hSCARB2 transgene. However, the correlation of virulence and viral loads in the CNS compartments or peripheral tissues like skin for HFMD or muscle for LP diseases in between B and C genotype of EV71 and CVA16 in our mice model will be further investigated. This fact was also shown in cynomolgus monkeys; application of 2 forms of EV71 BrCr, a temperature resistant strain (*tr*; grown at both 39.5°C and 35°C) isolated from patients with HFMD and encephalitis is more neurovirulent than a sensitive strain (*ts*; only grown at 35°C) isolated from cases with uncomplicated HFMD [15]. Certain genetic variations found in the genome of *tr* and *ts* strains might be related to neurovirulence [15]. Apart from the genetic variations in the EV71 genome, other factors might also determine susceptibility such as the age of the host that exhibits a different profile of gene expression in the cell. However, it may not be caused by the level of receptor expression; the expression of hSCARB2 was similar in newborns and adult Tg mice (Fig. 1D). Resolving these issues may help us to determine the severity of pathology in EV71 infection correlated with fatalities found in children less than 3 yrs of age [2] and in the experimental animal model.

We also compared the lethality of Tg mice while receiving different route of C genotype of EV71. i.p. administration of 3×10^6^ pfu of EV71 5746 to 7-d-old Tg mice induced with the severest LP and lethal results (6/6) as early as Day 4 post-infection, compared to 100% death (7/7) by Day 8 of s.c. administration (Fig. S4). Subcutaneous administration of EV71 induces either an HFMD-like syndrome or the deadly LP provided a broader time window, compared to the i.p. route for disease observation.

Immunization with active EV71 B4 and its vaccine (FI-E59) provided protection against the EV71 C2 challenge in hSCARB2-Tg mice because of induced neutralizing antibody as well as adaptive immune response (Fig. 7 and 8). However, innate immunity, particularly type I IFN, might be also involved for the phenotype because IFN-α/β levels in the spinal cord, and muscle of Tg mice during primary infection were upregulated and persisted through Day 7, the time of the EV71 challenge (data not shown). Administering the recombinant IFN-α protected the mice against infection with the mouse-adapted EV71. In contrast, infected mice treated with the anti-IFN-α antibody resulted in frequent deaths and higher tissue viral loads [48].

Corroborating these results, hSCARB2-Tg mice show greater susceptibility to natural strains B and C in orchestrating HFMD- and CNS-like syndromes than wild-type mice. Localized viral loads corresponding to pathological responses might induce secretion of pro-inflammatory mediators to exert protective or pathologic effects in the inflammatory lesions. hSCARB2-Tg mice may break through the limitation of current animal model applications that can prolong the time frame of mice age for the diseases induction and induce a unique HLS syndrome, and serve as an experimental model to study the pathology of EV71 infection and have potential for aiding the development of anti-EV71 therapeutic or prophylactic medicine.

## Materials and Methods

### Ethics Statement

All the experiments were conducted according to the guidelines of the Laboratory Animal Center of the National Health Research Institutes (NHRI), Taiwan. Animal use protocols have been reviewed and approved by the NHRI Institutional Animal Care and Use Committee (approved protocol no. NHRI-IACUC-099007-A).

### Cells and Viruses

Vero cells (Africa green monkey kidney cells) were cultured in a VP-SFM medium (Gibco) supplemented with 4 mM L-glutamine (Gibco) and the *RD cell* line, derived from a human rhabdomyosarcoma, was cultured in a DMEM medium with 10% fetal bovine serum (Gibco). They were maintained in a 37°C incubator equilibrated with 5% CO_2_. Four clinically isolated strains of EV71, E59 (B4) (GenBank: GQ150746.1), N2838-TW-03 (B5; GenBank: DQ008993.1), Tainan/5746/98 (C2) (GenBank: AF304457.1), and N3340-TW-02 (C4) strains (GenBank: EU131776.1), and one strain of CVA16, 5079 (GenBank: AF177911.1; obtained from Dr. Jen-Ren Wang, National Chen-Kung University, Tainan, Taiwan), was propagated in Vero cells based on the microcarrier cell culture bioprocess previously reported [49], [50]. The virus stocks were stored at −80°C. The titer of virus stocks was tested in a standard plaque-forming assay. Certified formalin-inactivated E59 (FI-E59) vaccine candidate containing sub-microgram of viral proteins formulated with alum adjuvant used in phase I clinical trial [35] was obtained from the co-correspondent author Dr. Pele Chong,

### Creation and Maintenance of hSCARB2-transgenic Mice

The human *SCARB2* gene (Genbank: NM_005506) was synthesized with codon optimization (Echo Chemic Ltd., Taiwan) and inserted into the pEF-1α plasmid modified from the background of pcDNA3.1(-) vector (Invitrogen), in which the cytomegalovirus enhancer/promoter was replaced by the promoter of elongation factor 1α (EF-1α) at the multiple cloning site using EcoRI and BamHI between the EF-1α promoter and bovine growth hormone (BGH) poly A tail to obtain the pEF-1α-hSCARB2 construct.

hSCARB2-Tg animals were generated by micro-injection of the endonuclease-linearized pEF-1α-hSCARB2 construct into fertilized C57BL/6 mouse embryos (National Applied Research Laboratories-Laboratory Animal Center, Taiwan) at the single-cell stage, which were subsequently implanted into pseudo-pregnant C57BL/6 female mice, generating transgenic animals following the protocol described previously [51]. Transgenic lineages were maintained by cross-mating hSCARB2-transgenic individuals to obtain inbred mice.

### Genotyping and Detection of hSCARB2 Gene Expression

Genomic DNA was isolated from mice tails using the Tissue genomic DNA extraction Mini kit (Favorgen). The human *SCARB2* transgene was detected in genomic DNA by a polymerase chain reaction (PCR) to amplify a 175 bp region using the specific primer set 1 (Table S1). The PCR condition was 95°C for 2 min; followed by 40 cycles of 95°C for 30 s, 50°C for 30 s, and 72°C for 10 s; followed by incubation at 72°C for 2 min.

To examine hSCARB2 expression, total RNA from the transgenic tissue of C57BL/6 mice using the TRIZOL reagent (Invitrogen) following the manufacturer instructions were isolated and subjected to quantitative RT-PCR reaction as following the protocol described in the method of Real-time RT-PCR section. Total RNA was converted into cDNA by the reaction of reverse transcription (RT) using random primers (Genomics BioSci&Tech) and reverse transcriptase (Bionovas). The primer set 2 was used and conditions used for PCR was 95°C for 3 min; followed by 40 cycles of 95°C for 10 s, 65°C for 20 s, and 72°C for 2 s; followed by incubation at 72°C for 2 min.

### Plaque Forming Assay

The plaque-forming assay for determining EV71 titer has been previously described [52]. The number of plaque-forming units (pfu) was calculated.

### Virus Neutralization Assay

Serum samples collected from immune mice were inactivated at 56°C for 30 min. Each sample was serially diluted two-fold with fresh cell-culture medium; then, 600 µL of a MOI = 1 of EV71 virus suspension, E59 or 5746 strain was added to each tube containing a 600 µL of 1∶10 initiated dilution of serially diluted serum. After incubation at 4°C for 1 h, the virus-serum mixtures were added to 6-well plates seeded with 5×10^5^ per well of RD cells and performed the plaque-forming assay. The 50% neutralization inhibition dose(ID50) was calculated as the reciprocal of the serum dilution that yielded a 50% reduction of the plaque formation that referenced to the plaque numbers generated in the tested wells infected with EV71 pre-incubated with 1∶10 diluted normal mouse serum. A mouse anti-EV71 Mab979 antibody (Chemicon International) was used as an internal positive control.

### EV71 Infection in hSCARB2-transgenic Mice

Tg or non-Tg (C57BL/6) mice were inoculated s.c. with EV71 E59 or 5746 strains, or VP-SFM medium alone. The mice were monitored daily for pathological signs, and were sacrificed at various times post-inoculation. The severity of HFMD and CNS syndromes was scored from 0 to 5 using the following criteria; for HFMD, 5 = 80% hair loss associated with scurf (HLS; white spots), 4 = >50% HLS, 3 = >30% HLS, 2 = >10% HLS, 1 = 10%–0% HLS, and 0 = no HLS on the back and abdomen. For scoring CNS diseases; 5 = severe front and rear limb paralysis (LP) and no movement, 4 = moderate 2 rear LP and hesitant movement, 3 = one rear LP with bending legs, 2 = mild rear limb bended, 1 = slightly rear limb bended, 0 = normal movement. LP is defined as the rigidness of mouse legs which are hesitate to move.

### Real-time RT-PCR

The synthesized cDNA was subjected to quantitative PCR analysis (the LightCycler® 480 SYBR Green Real-Time PCR system) using primer pairs specific to target genes. Mouse β-actin gene expression was detected as the internal control. The number of cycles required for amplification of the target gene was obtained, and the relative expression of the target gene was calculated as follows: the individual Ct obtained from the experimental group or control group was subtracted by its respective Ct (β-actin), and then 2^Normalized mean Ct (target gene from the tissue of Tg mouse without infection)^ was divided by 2^Normaliszed Ct (target gene from the tissue of EV71-infected Tg or non-Tg mouse)^. All primer sets were synthesized commercially by Genomics BioSci&Tech, Taiwan (Table S1).

### Immunohistochemical Staining

The tissues from sacrificed mice were rinsed in 10% buffered formalin (Sigma-Aldrich) and then embedded in paraffin (Sigma-Aldrich). Four micrometer sections were slided (Leica CM 1800) and placed on poly-L-lysine-coated glass slides before fixing with 3%–7% paraformaldehyde. The slides were incubated with the Mab979 antibody, isotype mouse IgG (Chemicon International, Inc.) or the anti-CD3 antibody (Dako Cytomation). Sections from uninfected Tg mice incubated with a primary antibody were included as negative control. The slides were washed and then added to the respective biotinylated antibody, followed by the Ultra-Sensitive ABC Mouse IgG staining Kit (Thermo Scientific). A red to brown peroxidase stain was developed using the DAB PLUS substrate kit (Zymed Laboratories). Bright field microscopy pictures were taken at 400X magnification.

### Enzyme-linked Immunosorbent Spot (ELISPOT) Assay

RBC-free 5×10^5^ per well of splenocytes were seeded into 96-well filtration plates (Millipore), pre-coated with antibodies for murine IL-4 or IFN-γ (eBioscience). The splenocytes were re-stimulated with heat-inactivated EV71 (viruses incubated at 80°C for 2 h), or Con A (positive control) or medium (negative control). The plates were kept in a 37°C incubator for 48 h before washing with PBS and adding to the corresponding biotinylated antibodies. The plates were washed, added to the streptavidin-alkaline phosphatase, and then incubated for 45 min. The plates were washed and developed by adding 3-amine-9-ethylcarbazole (Sigma-Aldrich) substrates in the dark before scoring the spots per well using the immunospot reader (C.T.L. IMMUNOSPOT, CELLULAR TECHNOLOGY LTD).

### Statistical Analysis

One-way anova with kruskal-Wallis test was used to analyze the statistic difference of the individual groups with HLS or LP symptoms. Logrank test was used to analyze the difference of survival rate in EV71-infected Tg vs. non-Tg mice. The unpaired t test with Welch’s correction statistic was used to analyze the difference of the tested gene expression and neutralizing antibody titer in between experimental groups. Results are considered statistically significance with a *p* value of <0.05. The symbols * and ** are used to indicate *p* values <0.05 and <0.01, respectively.

## Supporting Information

Figure S1 **Alignment of the gene sequence of human SCARB2 (hSCARB2) with mouse SCARB2 (mSCARB2).** Query of the hSCARB2 cDNA sequence was aligned to the subject of mSCARB2 cDNA sequence. The region of 126–151 and 369–389 nucleotides of hSCARB2 were highlighted to be targeted respectively by the forward and reverse primer set 2 (Table S1).
(TIF)

Figure S2 **Lethality of C2 genotype of EV71 in young mice.** Daily survival rate of 21-d-old hSCARB2-Tg or non-Tg mice injected with 3×10^6^ pfu of 5746 s.c. were monitored. The number (N) of mice per group was shown.
(TIF)

Figure S3 **Expression of pro-inflammatory cytokines in the peripheral tissues of EV71-infected mice.** RNA was extracted from the muscle (**A**) and spleen (**B**) of the hSCARB2-Tg and non-Tg mice on Day 7 post*-*s.c. injection with 3×10^4^ pfu of 5746, and then quantitative RT-PCR analysis was performed to quantify the expression of CXCL10, CCL3, TNF-α, IL-10, and IL-6. The relative gene expression was calculated as described in the legend of Fig. 6. A schematic representation of the target gene expression and the statistical average from 7 mice per group is shown. Unpaired student *t* test with Welch’s correction was used for statistical analysis.
(TIF)

Figure S4 **Lethal challenge of Tg mice with different routes of EV71 5746 strain.** Daily survival rate of 7-d-old hSCARB2-Tg mice injected i.p. or s.c. with 3×10^5^ pfu 5746 were monitored. The number (N) of mice per group was shown.
(TIF)

Table S1 List of the sequence of primer pairs specific to target genes.
(TIF)
