# Supplementary figures and images for: Human SCARB2 Transgenic Mice as an Infectious Animal Model for Enterovirus 71
Source: PLoS One. 2013 Feb 25;8(2):e57591. doi: 10.1371/journal.pone.0057591 (PMC3581494; doi:10.1371/journal.pone.0057591)

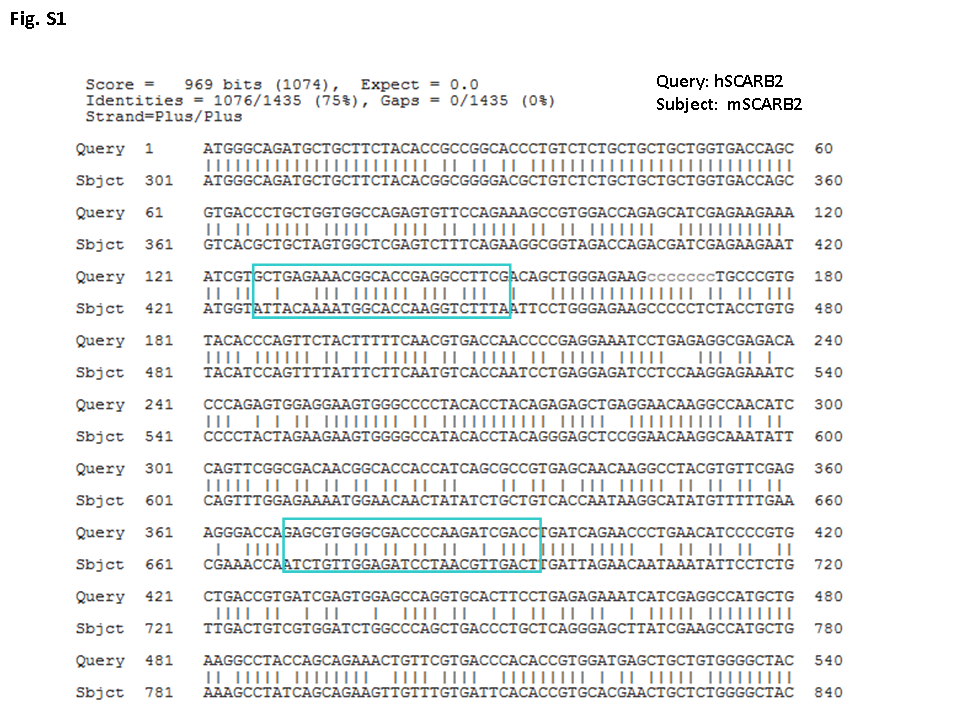

Supplement: Figure S1 — Alignment of the gene sequence of human SCARB2 (hSCARB2) with mouse SCARB2 (mSCARB2). Query of the hSCARB2 cDNA sequence was aligned to the subject of mSCARB2 cDNA sequence. The region of 126–151 and 369–389 nucleotides of hSCARB2 were highlighted to be targeted respectively by the forward and reverse primer set 2 (Table S1). (TIF) [file pone.0057591.s001.tif]
